# Blind Face Restoration *via* Multi-Prior Collaboration and Adaptive Feature Fusion

**DOI:** 10.3389/fnbot.2022.797231

**Published:** 2022-02-04

**Authors:** Zi Teng, Xiaosheng Yu, Chengdong Wu

**Affiliations:** Faculty of Robot Science and Engineering, Northeastern University, Shenyang, China

**Keywords:** blind face restoration, generative adversarial network, facial priors transformation, multi-prior collaboration, deep neural networks

## Abstract

Blind face restoration (BFR) from severely degraded face images is important in face image processing and has attracted increasing attention due to its wide applications. However, due to the complex unknown degradations in real-world scenarios, existing priors-based methods tend to restore faces with unstable quality. In this article, we propose a multi-prior collaboration network (MPCNet) to seamlessly integrate the advantages of generative priors and face-specific geometry priors. Specifically, we pretrain a high-quality (HQ) face synthesis generative adversarial network (GAN) and a parsing mask prediction network, and then embed them into a U-shaped deep neural network (DNN) as decoder priors to guide face restoration, during which the generative priors can provide adequate details and the parsing map priors provide geometry and semantic information. Furthermore, we design adaptive priors feature fusion (APFF) blocks to incorporate the prior features from pretrained face synthesis GAN and face parsing network in an adaptive and progressive manner, making our MPCNet exhibits good generalization in a real-world application. Experiments demonstrate the superiority of our MPCNet in comparison to state-of-the-arts and also show its potential in handling real-world low-quality (LQ) images from several practical applications.

## 1. Introduction

Face images are always one of the most popular types of images in our daily life, which record long-lasting precious memories and provide crucial information for identity analysis. Unfortunately, due to the limited conditions in the acquisition, storage and transmission devices, the degradations of face images are still ubiquitous in most real-world applications. The degraded face images not only impede human visual perception but also degrade face-related applications such as video surveillance and face recognition. This challenge motivates the restoration of high-quality (HQ) face images from the low-quality (LQ) face inputs which contain unknown degradations (e.g., blur, noise, compression), known as blind face restoration (BFR) (Chen et al., 2021; Wang et al., 2021; Yang et al., 2021). It has attracted increasing attention due to its wide applications.

Face images have face-specific geometry priors which include facial landmarks (Chen et al., 2018), facial parsing maps (Chen et al., 2018, 2021), and facial heatmaps (Yu et al., 2018). Therefore, many recent studies (Shocher et al., 2018; Zhang et al., 2018a, 2020; Soh et al., 2020) exploit extra face prior knowledge as inputs or supervision to recover accurate face shape and details. Benefiting from the incorporation of facial priors in deep neural networks (DNNs), these methods exhibit plausible and acceptable results on bicubic degraded faces. However, when applied to real-world scenarios, they are not applicable due to more complicated degradation. Additionally, the geometry priors estimated from LQ inputs contain very limited texture information for restoring facial details.

Other methods (Li et al., 2018, 2020b) investigate reference priors to generate realistic results. Reference priors can be only one face image, multiple face images, or facial component dictionaries, which can provide many identity-aware face details to the network. Nevertheless, when the identity of LQ is unavailable, the practical applications of referenced-based methods are limited. Additionally, the limited diversity and richness of facial component dictionaries also result in unrealistic restoration results.

Recently, with the rapid development of GAN techniques (Goodfellow et al., 2014), generative priors of pretrained face GAN models, such as StyleGAN (Karras et al., 2019, 2020), are exploited for real-world face restoration (Gu et al., 2020; Menon et al., 2020; Pan et al., 2021). Since face synthesis GANs can generate visually realistic faces with rich and diverse details, it is reasonable to incorporate such generative priors into the face restoration process. These methods first map the LQ input image to an intermediate latent code, which then controls the pretrained GAN at each convolution layer to provide generative priors such as facial textures and colors. This, however, leads to unstable quality of restored faces when dealing with the LQ face image. Due to the low-dimension of latent codes, such a decoupling control method is insufficient to guide the precise restoration process.

Another category of approaches involves performing degradation estimation (Michaeli and Irani, 2013; Bell-Kligler et al., 2019) to provide degradation information for the conditional restoration of LQ face images with unknown degradations. Although this design incorporates human knowledge about the degradation process and implies a certain degree of interpretability, the degradation process in the real world is too complex to be estimated, which fails to bring degradation estimation into full play.

In this article, we investigate the problem of BFR and aim at restoring HQ faces from LQ inputs with complicated degradation. For achieving a better trade-off between realness and fidelity, we propose a multi-prior collaboration network (MPCNet) to seamlessly integrate the advantages of generative priors and face-specific geometry priors. To be specific, we first pretrain an HQ face synthesis GAN and a parsing mask prediction network, and then embed them into a U-shaped DNN as decoder priors to guide face restoration. On the one hand, the encoder part of U-shaped DNN learns to map the LQ input to an intermediate latent space for global face reproduction, which then controls the generator of face synthesis GAN to provide the desired generative priors for HQ face images restoration. On the other hand, the decoder part of U-shaped DNN leverages the encoded intermediate spatial features and diverse facial priors to restore the HQ face in a progressive manner, during which the generative priors can provide adequate details and the parsing map priors provide geometry and semantic information. Instead of direct concatenation, we proposed multi-scale adaptive priors feature fusion (APFF) blocks to incorporate the prior features from pretrained face synthesis GAN and face parsing network in an adaptive and progressive manner. In each APFF block, we integrate generative priors and parsing maps priors with decoded facial features to generate the fusion feature maps for guiding face restoration. In this way, when applying to complicated degradation scenarios, the fusion feature maps can correctly find where to incorporate guidance prior features in an adaptive manner, making our MPCNet exhibits good generalization in a real-world application. The main contributions of this study include:

- We propose a MPCNet to seamlessly integrate the advantages of generative priors and face-specific geometry priors. We pretrain an HQ face synthesis GAN and a parsing mask prediction network, and then embed them into a U-shaped DNN as decoder priors to guide face restoration, during which the generative priors can provide adequate details and the parsing map priors provide geometry and semantic information.
- We propose an APFF block to incorporate the prior features from pretrained face synthesis GAN and face parsing network in an adaptive and progressive manner, making our MPCNet exhibits good generalization in a real-world application.
- Experiments demonstrate the superiority of our MPCNet in comparison to state-of-the-arts, and show its potential in handling real-world LQ images from several practical applications.

## 2. Related Study

**Facial geometry prior knowledge**: Face images have face-specific geometry prior information, which includes 3D facial prior, facial landmarks, face depth map, facial parsing maps, and facial heatmaps. To recover facial images with much clearer facial structure, researchers begin to utilize facial prior knowledge to design the effective face restoration network. Song et al. (2017) proposed to utilize a pre-trained network to extract facial landmarks to divide facial components and feed the five components into different branches to recover corresponding components. Jiang et al. (2018) developed a DNN denoiser and multi-layer neighbor component embedding for face restoration, which first recovered the global face images and then compensated missing details for every component. Wang et al. (2020) proposed the parsing map guided multi-scale attention network to extract the parsing map from LQ and then fed the concatenation of the parsing map and LQ into the subnetworks to produce HQ results. Supposed that the depth map could provide geometric information, Fan et al. (2020) built a subnetwork to learn the depth map from LR and then imported depth into the HQ network to facilitate the facial reconstruction. Benefiting from the incorporation of facial priors in DNNs, these methods exhibit plausible and acceptable results on bicubic degraded faces. However, when applied to real-world scenarios, they are not applicable due to more complicated degradation. Additionally, the geometry priors estimated from LQ inputs contain very limited texture information for restoring facial details. Since face synthesis GANs can generate visually realistic faces with rich and diverse details, it is reasonable to incorporate such generative priors into the face restoration process.

**Figure 1:**
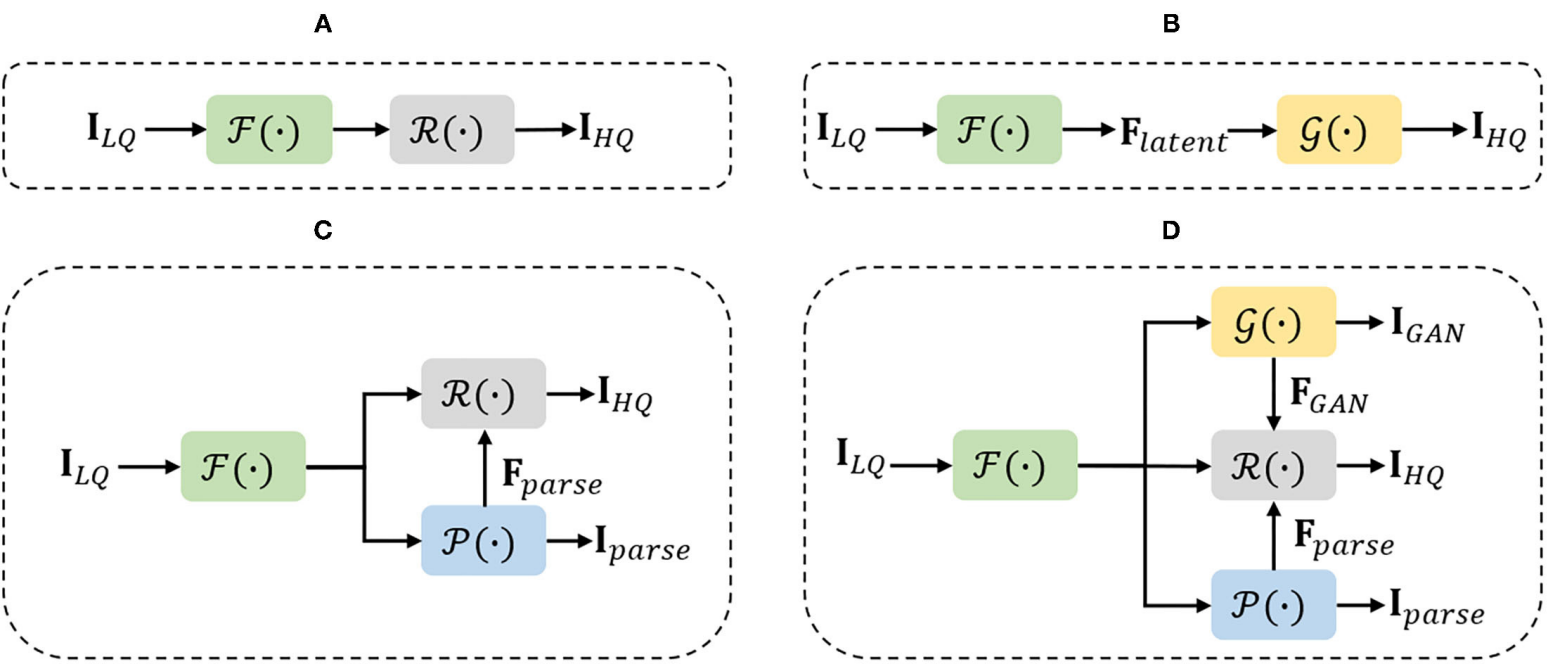
Diagrams of the main ideas of four different paradigms applying for blind face restoration (BFR). In the figures, F(·), G(·), P(·), and R(·) denote the feature extraction, generative adversarial network (GAN) prior production, Parsing map prior production, and the restoration network, respectively. **(A)** Framework of basic CNN-based methods. **(B)** Framework of GAN prior embedded network. **(C)** Framework of parsing map guided network. **(D)** Framework of our multi-prior collaboration network.

**Facial generative prior knowledge**: Recently, with the rapid development of GAN techniques (Goodfellow et al., 2014), generative priors of pretrained face generative adversarial network (GAN) models, such as StyleGAN (Karras et al., 2019, 2020), are exploited for real-world face restoration (Gu et al., 2020; Menon et al., 2020; Pan et al., 2021). Generative Priors of pretrained GANs (Karras et al., 2017, 2019, 2020; Brock et al., 2018) are previously exploited by GAN inversion (Abdal et al., 2019; Gu et al., 2020; Zhu et al., 2020; Pan et al., 2021), whose primary aim is to map the LQ input image to an intermediate latent code, which then controls the pretrained GAN at each convolution layer to provide generative priors such as facial textures and colors. Yang et al. (2021) proposed to embed the GAN prior learned for face generation into a DNN for face restoration, then jointly fine-tuned the GAN prior network with the DNN. Therefore, the latent code and noise input can be well generated from the degraded face image at different network layers. Wang et al. (2021) proposed to utilize the rich and diverse generative facial priors that contained sufficient facial textures and color information to restore the LQ face images. However, extensive experiments have shown that, due to the low-dimension of latent codes, such decoupling control method is insufficient to guide the precise restoration process and leads to unstable quality of restored faces when dealing with the LQ face image. For achieving a better trade-off between realness and fidelity, we rethink the characteristic of the BFR task and turn to the direction of incorporating various types of facial priors for recovering HQ faces. To that end, we propose a novel multi-prior collaboration framework to seamlessly integrate the advantages of generative priors and face-specific geometry priors, which shows its potential in handling real-world LQ images from several practical applications (see Figure 1). For preserving high fidelity, we reform the GAN blocks in StyleGANv2 by removing the noise inputs to avoid the generation of extra stochastic facial details. Then, we design an APFF block to incorporate the prior features from pretrained face synthesis GAN and face parsing network in an adaptive and progressive manner. In general, our main contribution is to explore the solution of the BFR task from a different perspective and provide an effective method that can achieve promising performance on both synthetic and real degraded images.

## 3. Methodology

In this section, we first describe the degradation model and our framework in detail, then introduce the adaptive prior features fusion, and finally give the learning objectives used to train the whole network.

### 3.1. Problem Formulation

To tackle severely degraded faces in real-world scenarios, the training data is synthesized by a complicated degradation that can be formulated as follows:


(1) $$\begin{array}{l} \text{x}={\left[\left(\text{y}⊛{\text{k}}_{\sigma }\right){↓}_{r}+{\text{n}}_{\delta }\right]}_{{JPEG}_{q}} \end{array}$$

where **x** is the LQ face, **y** is the HQ face image, **k**_σ_ is a blur kernel, ⊛ denotes convolution operation, ↓_*r*_ represents the standard **r**-fold downsampler, **n**_δ_ refers to the Gaussian noise with SD δ, and the *JPEG*_*q*_ denotes the JPEG compression operator with a quality factor *q*. In our implementation, for each training pair, we randomly select the blur kernel **k** from the following four kernels: Gaussian Blur (3 ≤ σ ≤ 15), Average Blur (3 ≤ σ ≤ 15), Median Blur (3 ≤ σ ≤ 15), and Motion Blur (5 ≤ σ ≤ 25). The scale factor *r* is randomly sampled from [4:16]. The addictive white gaussian noise (AWGN) **n**_δ_ is sampled channel-wise from a normal distribution with (0 ≤ δ ≤ 0.1 × 255). The compression level *q* is randomly sampled from [10:65], where higher means stronger compression and lower image quality.

### 3.2. Overview of MPCNet

**Figure 2:**
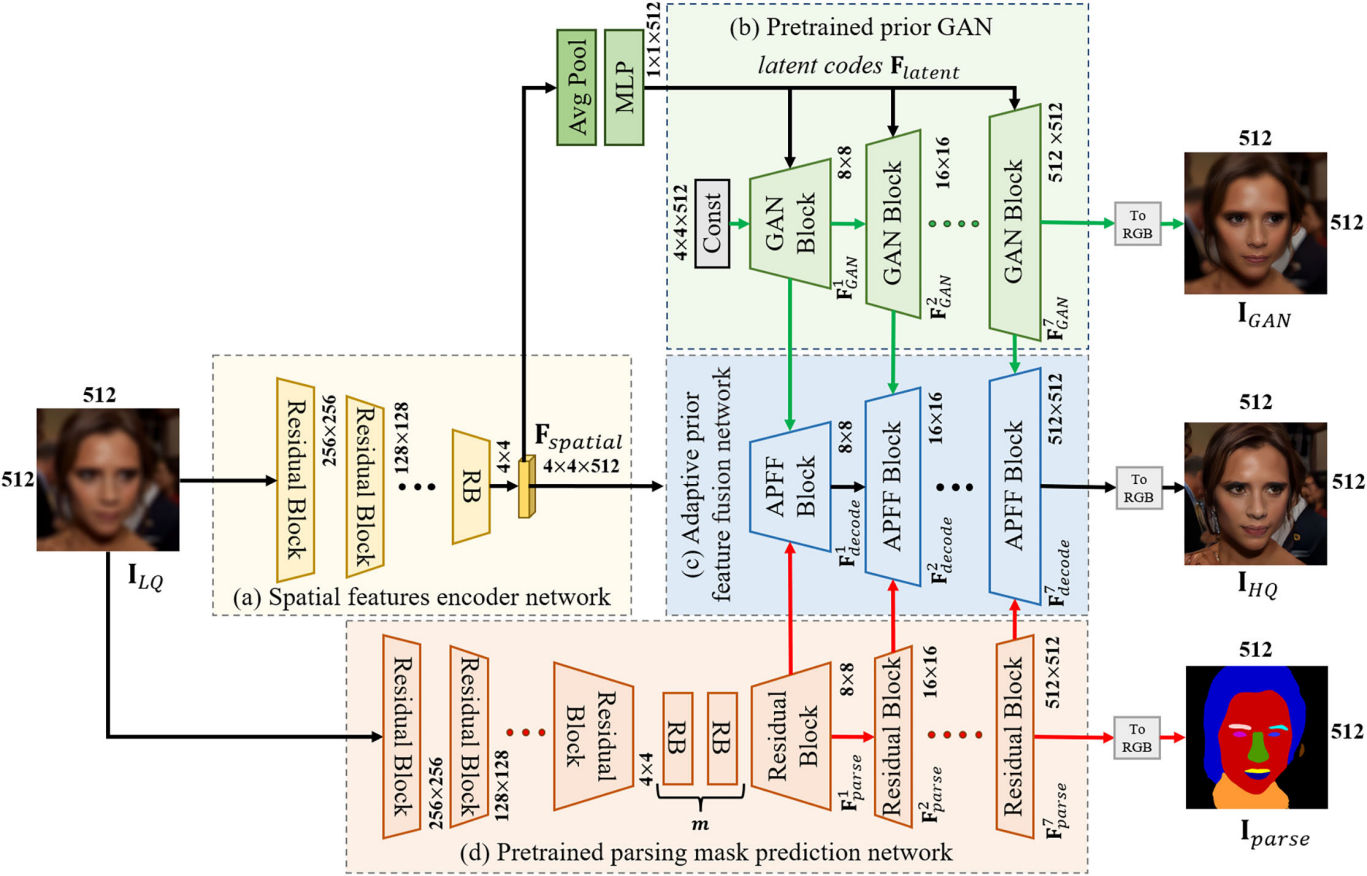
The detailed architecture of the proposed network. **(a)** Spatial feature encoder network. **(b)** Pretrained prior GAn. **(c)** Adaptive prior feature fusion network. **(d)** Pretrained parsing mask prediction network.

To begin with, BFR is defined as the task of reconstructing the HQ face image **y** from an LQ input facial image **x** suffering from unknown degradation. Figure 2 illustrates the overall framework of the proposed MPCNet consisting of spatial features encoder network, adaptive prior fusion network, pretrained face synthesis GAN, and pretrained parsing mask prediction network.

#### 3.2.1. U-Shape Backbone Network

The backbone of our MPCNet is composed of the spatial features encoder network and adaptive prior fusion decoder network. It starts with a degraded face image **I**_*LQ*_ of size 512 × 512 × 3. When the input is of a different size, we simply resize it to 512 × 512 with bicubic sampling. Then, **I**_*LQ*_ goes through several down-sample residual groups to generate an intermediate latent space *W* which is shared by adaptive prior fusion decoder network and pretrained face synthesis GAN (such as StyleGANv2; Karras et al., 2020). To progressively fuse the decoded spatial features and multiple priors, we present the APFF blocks to construct the decoder part of the U-shape backbone network. The feature ${\text{F}}_{decode}^{7}$ from the last APFF block is passed on to a single ToRGB convolution layer and predicts the final output **I**_*HQ*_. More details about the APFF block will be given in the next section.

#### 3.2.2. Pretrained Face Synthesis GAN

Due to the high capability of GANs in generating HQ face images, we leverage pretrained StyleGAN2 prior to providing diverse and rich facial details for our BFR task. To utilize the generative priors, previous methods typically map the input image to its closest latent codes *Z* and then generate the corresponding output directly. However, due to the low-dimension of latent codes, such decoupling control method is insufficient to guide the precise restoration process and leads to unpredictable failures. Instead of generating the final HQ face image directly, we propose to exploit the intermediate convolutional features of pretrained GAN as priors and further combine them with other types of priors for better fidelity.


(2) $$\begin{array}{l} {\text{F}}_{latent},{\text{F}}_{spatial}=Unet\left(\text{x}\right) \end{array}$$

Specifically, given the encoded intermediate spatial features **F**_*spatial*_ of the input image (produced by the encoder part of the U-shape backbone network, Equation 2), we first map it to the latent codes **F**_*spatial*_ with global pooling operation and several multi-layer perceptron layers (MLP). The latent codes **F**_*latent*_ then pass through each convolution layer in the pretrained GAN and generate GAN features for each resolution scale.


(3) $$\begin{array}{l} {\text{F}}_{latent}=MLP\left({\text{F}}_{spatial}\right),{\text{F}}_{GAN}=StyleGAN\left({\text{F}}_{latent}\right), \end{array}$$

**Figure 3:**
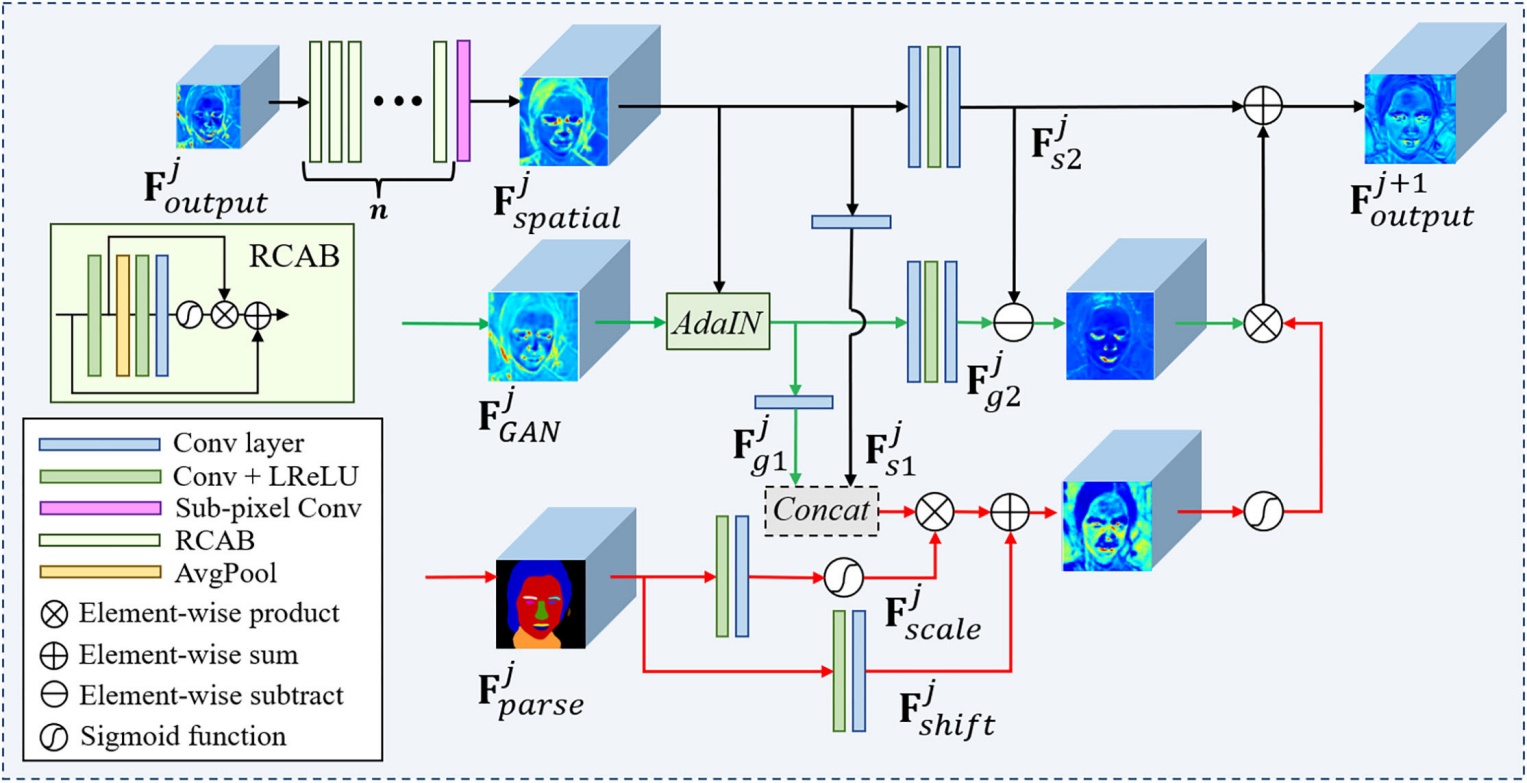
The detailed structures of a GAN block. The definition of “Mod” and “Demod” can be found in Karras et al. (2020).

**Figure 4:**
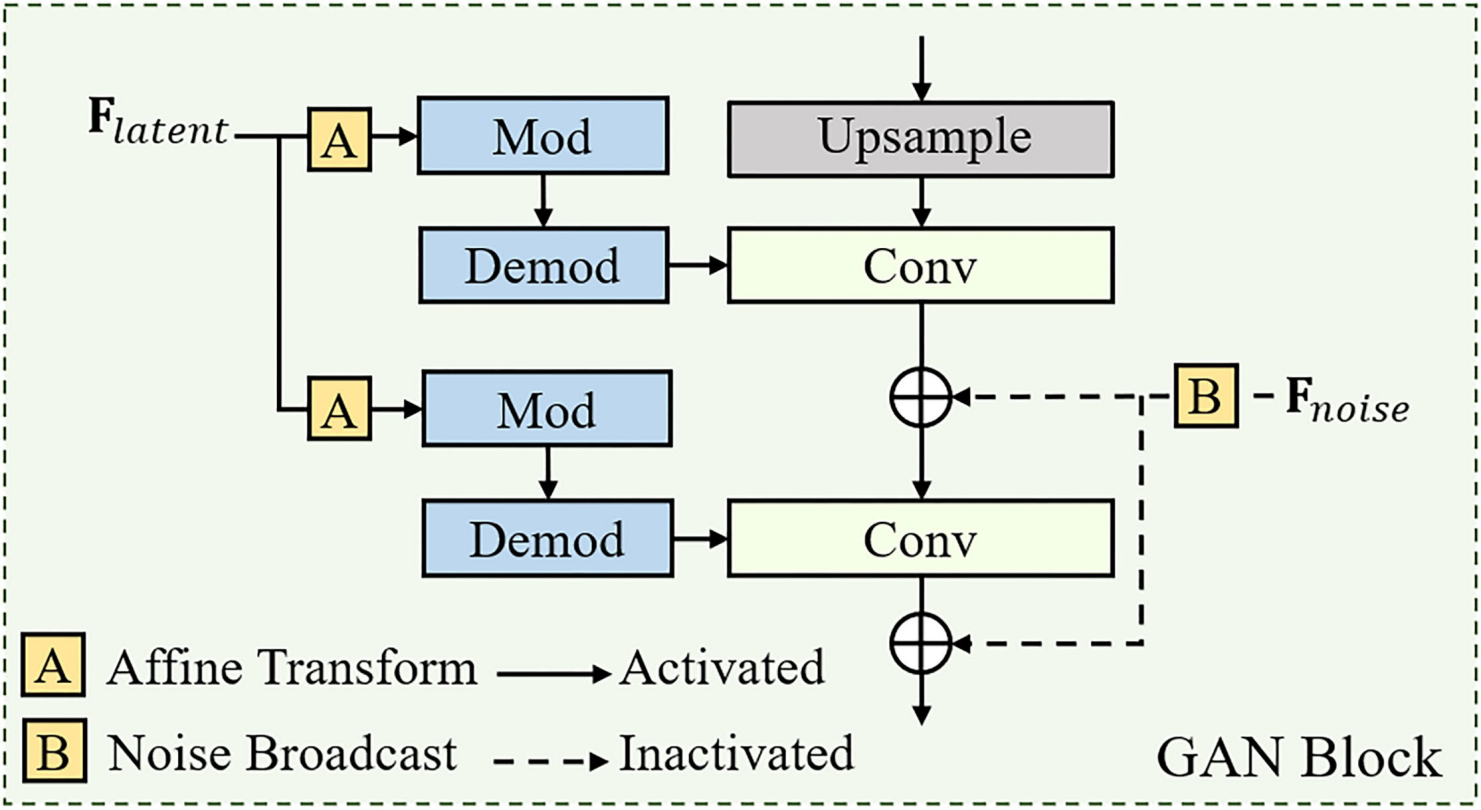
The detailed structures of the adaptive prior feature fusion (APFF) block. The cascading residual channel attention blocks (RCABs) (Zhang et al., 2018c) can make the feature extraction focus on more informative components of the LQ features.

The structure of the GAN block is shown in Figure 3, which is consistent with the architecture in StyleGANv2. Additionally, the number of GAN blocks is equal to the number of APFF blocks in the U-shape backbone network, which is related to the resolution of the input face image. For the realness of the synthetic face, the original StyleGANv2 generates stochastic detail by introducing explicit noise inputs. However, the reconstructed HQ face image is required to faithfully approximate the ground-truth face image. For achieving a better trade-off between realness and fidelity, we abandon the noise inputs for all GAN blocks (see Figure 4).

#### 3.2.3. Pretrained Parsing Mask Prediction Network

To further improve the fidelity of the restored face image, we pretrain a parsing mask prediction network to provide the geometry and semantic information for covering the deficiencies of GAN priors. As illustrated in Figure 2D, since learning the mapping from LQ→parsing maps is much simpler than face restoration, the parsing mask prediction network only employs an encoder-decoder framework. It begins with 7 downsample residual blocks, followed by 10 residual blocks, and 7 upsample residual blocks. The last feature ${\text{F}}_{parse}^{7}$ is passed on to a single ToRGB convolution layer and predicts the final output **I**_*parse*_. Besides, we conduct extensive experiments to demonstrate the robustness of the parsing mask prediction network on LQ face images with unknown degradations.

### 3.3. Adaptive Feature Fusion

It is extremely complex to recover HQ faces from the LQ counterparts in real-world scenarios, due to the complicated degradation, diverse poses, and expressions. Therefore, it is natural to consider to combining the different facial priors and let them collaborate to improve the reconstruction quality. Since each facial prior has its shortcomings especially for a specific application, we propose a novel collaboration module that combines multiple facial priors, in which the feature translation, transformation, and fusion are considered for improving the restoration performance and generalization ability of our MPCNet. The APFF block is designed to integrate generative priors ${\text{F}}_{GAN}^{j}$ and parsing maps priors ${\text{F}}_{parse}^{j}$ with decoded facial features ${\text{F}}_{spatial}^{j}$ to generate the fusion feature maps ${\text{F}}_{output}^{j+1}$ for guiding face restoration. The rich and diverse details provided by ${\text{F}}_{GAN}^{j}$ can greatly alleviate the difficulty of degradation estimation and image restoration. However, due to the deficiency of the decoupling control method in StyleGANv2, the style condition of ${\text{F}}_{GAN}^{j}$ is unstable and inconsistent with ${\text{F}}_{spatial}^{j}$, which should be considered before feature fusion.

**AdaIN**. AdaIN (Huang and Belongie, 2017) is first proposed to translate the content features to the desired style. Due to its efficiency and compact representation (Karras et al., 2020), AdaIN is adopted to adjust ${\text{F}}_{GAN}^{j}$ to have a similar style condition with the restored feature of degraded image. The AdaIN operation can be formulated as:


(4) $$\begin{array}{l} AdaIN\left({\text{F}}_{GAN}^{j},{\text{F}}_{spatial}^{j}\right)=\sigma \left({\text{F}}_{spatial}^{j}\right)\frac{{\text{F}}_{GAN}^{j}-\mu \left({\text{F}}_{GAN}^{j}\right)}{\sigma \left({\text{F}}_{GAN}^{j}\right)}\\ \;+\mu \left({\text{F}}_{spatial}^{j}\right),\\ \;{\text{F}}_{g1}^{j}=f_{conv1}\left[AdaIN\left({\text{F}}_{GAN}^{j},{\text{F}}_{spatial}^{j}\right)\right],\\ \;{\text{F}}_{g2}^{j}=f_{conv2}\left[AdaIN\left({\text{F}}_{GAN}^{j},{\text{F}}_{spatial}^{j}\right)\right], \end{array}$$

where σ(·) denotes the mean operation and μ(·) denotes the SD operation. With AdaIN operation, ${\text{F}}_{GAN}^{j}$ can, thus, be aligned with ${\text{F}}_{spatial}^{j}$ by style condition such as color, contrast, and illumination. Intermediate generative features ${\text{F}}_{g1}^{j}$ and ${\text{F}}_{g2}^{j}$ are generated by *f*_*conv*1_(·) and *f*_*conv*2_(·) which denote 3 × 3 convolutions and are exploited to reduce the channel numbers and refine features, respectively. Besides, the intermediate spatial features ${\text{F}}_{s1}^{j}$ and ${\text{F}}_{s2}^{j}$ are also generated from ${\text{F}}_{spatial}^{j}$ by the same process.

**Spatial feature transform**. Motivated by the observation that GAN priors are incapable to capture the geometry information of the overall face structure due to the decoupling control method, we propose to exploit the parsing map prior to providing the geometry and semantic information for covering the shortage of GAN priors. Specifically, we introduce the guidance features ${\text{F}}_{guide}^{j}$ to direct the fusion process of ${\text{F}}_{GAN}^{j}$ and ${\text{F}}_{spatial}^{j}$. Additionally, the generation of ${\text{F}}_{guide}^{j}$ considers the ${\text{F}}_{GAN}^{j}$, ${\text{F}}_{spatial}^{j}$, and ${\text{F}}_{parse}^{j}$. For spatial-wise feature modulation, we employ Spatial Feature Transform (SFT), named *SFT*(·), Wang et al. (2018b) to generate the affine transformation parameters with ${\text{F}}_{parse}^{j}$. At each resolution scale, the *SFT*(·) learns a mapping function *f*(·) that provides a modulation parameter pair **α**, **β** according to the parsing maps ${\text{F}}_{parse}^{j}$, and then utilities **α**, **β** to provide spatially fine-grained control to the concatenation of ${\text{F}}_{GAN}^{j}$ and ${\text{F}}_{spatial}^{j}$.


(5) $$\begin{array}{l} \left(\alpha ,\beta \right)=f\left({\text{F}}_{parse}^{j}\right), \end{array}$$

The concatenation of ${\text{F}}_{GAN}^{j}$ and ${\text{F}}_{spatial}^{j}$ is modified by scaling and shifting feature maps according to the transformation parameters:


(6) $$\begin{array}{l} {\text{F}}_{guide}^{j}=SFT\left(Concat\left[{\text{F}}_{g1}^{j},{\text{F}}_{s1}^{j}\right]|\alpha ,\beta \right)=\alpha ⊗Concat\left[{\text{F}}_{g1}^{j},{\text{F}}_{s1}^{j}\right]+\beta , \end{array}$$

where *Concat*[;] denotes the concatenation operation and $Concat\left[{\text{F}}_{g1}^{j},{\text{F}}_{s1}^{j}\right]$ denotes the concatenated feature maps, which have the same dimension with **α** and **β**, and ⊗ indicates element-wise multiplication.

On the one hand, the facial generative priors generally contain HQ facial texture details. On the other hand, the facial parse priors have more shape and semantic information and, thus, are more reliable for the global facial region. Considering that ${\text{F}}_{GAN}^{j}$ and ${\text{F}}_{parse}^{j}$ can mutually convey complementary information for each other, we combine them for better reconstruction of the HQ face image. We first calculate the errors between generative features and spatial features to highlight the inconsistent facial components that need correction. Then we exploit a gating module *softmax*(·) to generate the semantic-guided map from parse features. Finally, we combine the semantic-guided maps and the feature of inconsistent facial components to refine the initial spatial features in early layers for obtaining better results. The output of each APFF block can be written as,


(7) $$\begin{array}{l} {\text{F}}_{output}^{j+1}=\left({\text{F}}_{g2}^{j}-{\text{F}}_{s2}^{j}\right)⊗softmax\left({\text{F}}_{guide}^{j}\right)+{\text{F}}_{s2}^{j} \end{array}$$

As a result, this helps to make full use of the rich and diverse texture information from ${\text{F}}_{GAN}^{j}$ as well as shape and semantic guidance from ${\text{F}}_{parse}^{j}$ in an adaptive manner, thereby achieving a good balance between realness and faithfulness. Besides, we conduct APFF block at each resolution scale to facilitate progressive fusion and finally generate the restored face. In this way, when applying to complicated degradation scenarios, the fusion feature maps can correctly find where to incorporate guidance prior features in an adaptive manner, making our MPCNet exhibits good generalization in a real-world application.

### 3.4. Learning Objective

For achieving a better trade-off between realness and fidelity, following previous BFR methods (Chen et al., 2018; Wang et al., 2018a,c; Li et al., 2020a,b), we apply 1) reconstruction loss that constrains the outputs to faithfully approximate to the ground-truth face image, 2) adversarial loss that generates the visually realistic details for the photo-realistic face restoration, and 3) gram matrix loss that helps in better synthesize texture details.

**Reconstruction loss**. We combine the pixel and feature space mean square error (MSE) to constrain the network output ${\hat{\text{I}}}_{HQ}$ close to the ground truth **I**_*HQ*_. As shown in follows, the second term is perceptual loss (Yu and Porikli, 2017; Wang et al., 2018b):


(8) $$\begin{array}{l} L_{rec}=\lambda_{MSE}∥{\text{I}}_{HQ}-{\hat{\text{I}}}_{HQ}{∥}_{1}+\lambda_{perc}\overset{4}{\underset{i=1}{\sum }}∥\varphi_{i}\left({\text{I}}_{HQ}\right)-\varphi_{i}\left({\hat{\text{I}}}_{HQ}\right){∥}_{1}, \end{array}$$

where φ_*i*_(·) represents the features from the *i*-th layer of the pretrained VGGFace model (Cao et al., 2018). λ_*MSE*_ and λ_*perc*_ denote the trade-off loss weights parameters. In this study, we set *i* ∈ [1, 2, 3, 4].

**Adversarial loss**. Adversarial loss has been proved to be an effective and critical method in improving visual quality. In both generator and discriminator, we incorporate spectral normalization (Miyato et al., 2018) on the weights of each convolution layer to stabilize the learning. Furthermore, we adopt the hinge version of adversarial loss as the objective function (Brock et al., 2018; Zhang et al., 2019), defined as:


(9) $$\begin{array}{l} L_{adv,D}=𝔼\left[max\left(0,1-D\left({\text{I}}_{HQ}\right)\right)\right]+𝔼\left[max\left(0,1+D\left({\hat{\text{I}}}_{HQ}\right)\right)\right],\\ L_{adv,G}=-𝔼\left[D\left({\hat{\text{I}}}_{HQ}\right)\right] \end{array}$$

In this study, $L_{adv,D}$ is used to update the discriminator, while $L_{adv,G}$ is adopted to update the MPCNet for blind face restoration.

**Gram matrix loss**. Gram matrix loss (Gatys et al., 2016) has demonstrated that style transfer helps a lot in synthesizing visually plausible textures. We use pretrained VGGFace (Cao et al., 2018) features of layer relu2_1, relu3_1, relu4_1, and relu5_1 to calculate gram matrix loss, which is formulated as:


(10) $$\begin{array}{l} L_{style}=\overset{4}{\underset{i=1}{\sum }}\frac{∥\varphi_{i}{\left({\text{I}}_{HQ}\right)}^{T}\varphi_{i}\left({\text{I}}_{HQ}\right)-\varphi_{i}{\left({\hat{\text{I}}}_{HQ}\right)}^{T}\varphi_{i}\left({\hat{\text{I}}}_{HQ}\right){∥}^{2}}{C_{i}H_{i}W_{i}}, \end{array}$$

where φ_*i*_(·) represents the features from the *i*-th layer of the pretrained VGGFace model.

## 4. Experiment Results

### 4.1. Dataset and Experimental Settings

**Figure 5:**
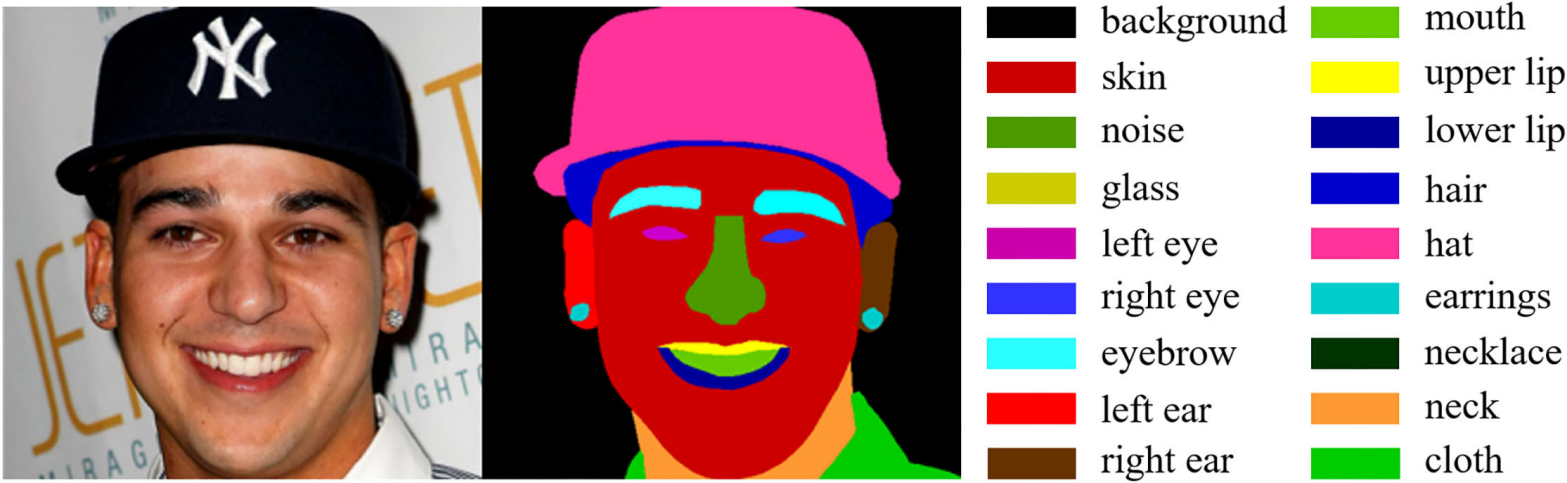
A visual example of facial parsing map information.

**Training datasets**. We first adopt the CelebA-Mask-HQ (Lee et al., 2020) to pre-train the face parsing mask prediction network, which contains 30,000 HQ face images with a size of 1, 024 × 1, 024 pixels. As shown in Figure 5, each image of CelebA-Mask-HQ has a segmentation mask of facial attributes. To build the training set, we randomly choose 24,000 HQ images and resize all images to 512 × 512 pixels as ground-truth. Similar to Li et al. (2020a), we adopt the degradation model in section Problem formulation with randomly sampled parameters to synthesize the corresponding LQ images. Then we adopt the FFHQ dataset (Karras et al., 2019) to train the GAN prior network and the final MPCNet. FFHQ dataset contains 70,000 HQ face images with a size of 1, 024 × 1, 024 pixels. In the same way as CelebA-Mask-HQ, we synthesize the LQ inputs with Equation (1) during training.

**Testing datasets**. We construct one synthetic test dataset and one real-world LQ test dataset to validate the ability of the proposed method on handling the BFR. Additionally, all these test datasets have no overlap with the training datasets. For the synthetic test dataset, we first randomly choose 3,000 HQ images from the CelebA-HQ dataset (Karras et al., 2017). Then the generation way of testing pairs is the same as the training dataset, namely CelebA-Test. For the real LQ test dataset, we collect 1,000 LQ faces from CelebA (Liu et al., 2015) and 500 old photos from the web. We coarsely crop square regions in each image according to their face regions and resize them to 512 × 512 pixels using bicubic upsampling. In the end, we put all these images together and generate the real LQ test dataset containing 1,500 real LQ faces, namely **Real-Test**.

**Implementation**. We adopt Adam optimizer (Kingma and Ba, 2014) with δ1 = 0.9, δ2 = 0.99, and ε = 10^−8^ to train our MPCNet with a batch size of 8. During training, we augment the training images by randomly horizontally flipping. The learning rate is initialized as 2 * 10^−4^ and then decreased to half when the reconstruction loss is no longer dropping on the validation set. Our proposed model is implemented on the Pytorch framework using two NVIDIA RTX 2080Ti GPUs.

### 4.2. Evaluation Index

For synthetic test datasets with ground truth, two widely used image quality assessment indexes, peak signal-to-noise ratio (PSNR) (Hore and Ziou, 2010) and structural similarity (SSIM) (Wang et al., 2004), are used as the criteria for evaluating the performance of models, which are defined as follows:


(11) $$\begin{array}{l} MSE\left(\text{x},\text{y}\right)=\sqrt{\frac{1}{n}\overset{n}{\underset{i=1}{\sum }}{\left(x_{i}-y_{i}\right)}^{2}} \end{array}$$

where **x** is the target image; **y** is the HQ image which is generated from the LQ image; *x*_*i*_ and *y*_*i*_ represent the values of *i* − *th* pixel in **x** and **y**, respectively, and *n* denotes the pixel number in the image. Then we calculate the PSNR as follows:


(12) $$\begin{array}{l} PSNR\left(\text{x},\text{y}\right)=10\cdot \log_{10}\frac{MAX^{2}}{MSE\left(\text{x},\text{y}\right)} \end{array}$$

where *MAX* denotes the maximum possible pixel value of the image. It is set to 255 in our experiments since the pixels of the images are represented using 8 bits per sample. PSNR is used to evaluate the performance of the proposed method in reconstructing HQ images. Instead of measuring the error between the ground-truth HQ image and the reconstructed HQ image, Wang et al. (2004) proposed an image quality assessment metric called SSIM to compute the SSIM of two images, and the SSIM value of the reconstructed HQ image **y** is computed as follows:


(13) $$\begin{array}{l} SSIM\left(\text{x},\text{y}\right)=\frac{\left(2\mu_{\text{x}}\mu_{\text{y}}+C_{1}\right)\left(2\sigma_{\text{x}\text{y}}+C_{2}\right)}{\left(\mu_{\text{x}}^{2}+\mu_{\text{y}}^{2}+C_{1}\right)\left(\sigma_{\text{x}}^{2}+\sigma_{\text{y}}^{2}+C_{2}\right)} \end{array}$$

where μ_**x**_, μ_**y**_, σ_**x**_, σ_**y**_, and σ_**xy**_ represent the local means, SDs, and cross-covariance for images **x** and **y**, respectively. $C_{1}={\left(k_{1}L\right)}^{2}$ and $C_{2}={\left(k_{2}L\right)}^{2}$ are variables to stabilize the division with a weak denominator, where *L* is the dynamic range of the pixel values that are set to 255 and *k*_1_ and *k*_2_ are set to 0.01 and 0.03 in our experiments.

Besides, since pixel space metrics are only based on local distortion measurement and inconsistent with human perception, the Learned Perceptual Image Patch Similarity (LPIPS) score (Zhang et al., 2018b) is adopted to evaluate the perceptual realism of generated faces. For a real LQ test dataset without ground truth, the widely-used non-reference perceptual metrics: Fréchet Inception Distances (FID) (Heusel et al., 2017) is used as the criteria for evaluating the performance of the models. We choose 3,000 HQ images from the CelebA-HQ dataset as the reference dataset to evaluate the results of the real LQ test dataset.

### 4.3. Ablation Study

**Figure 6:**
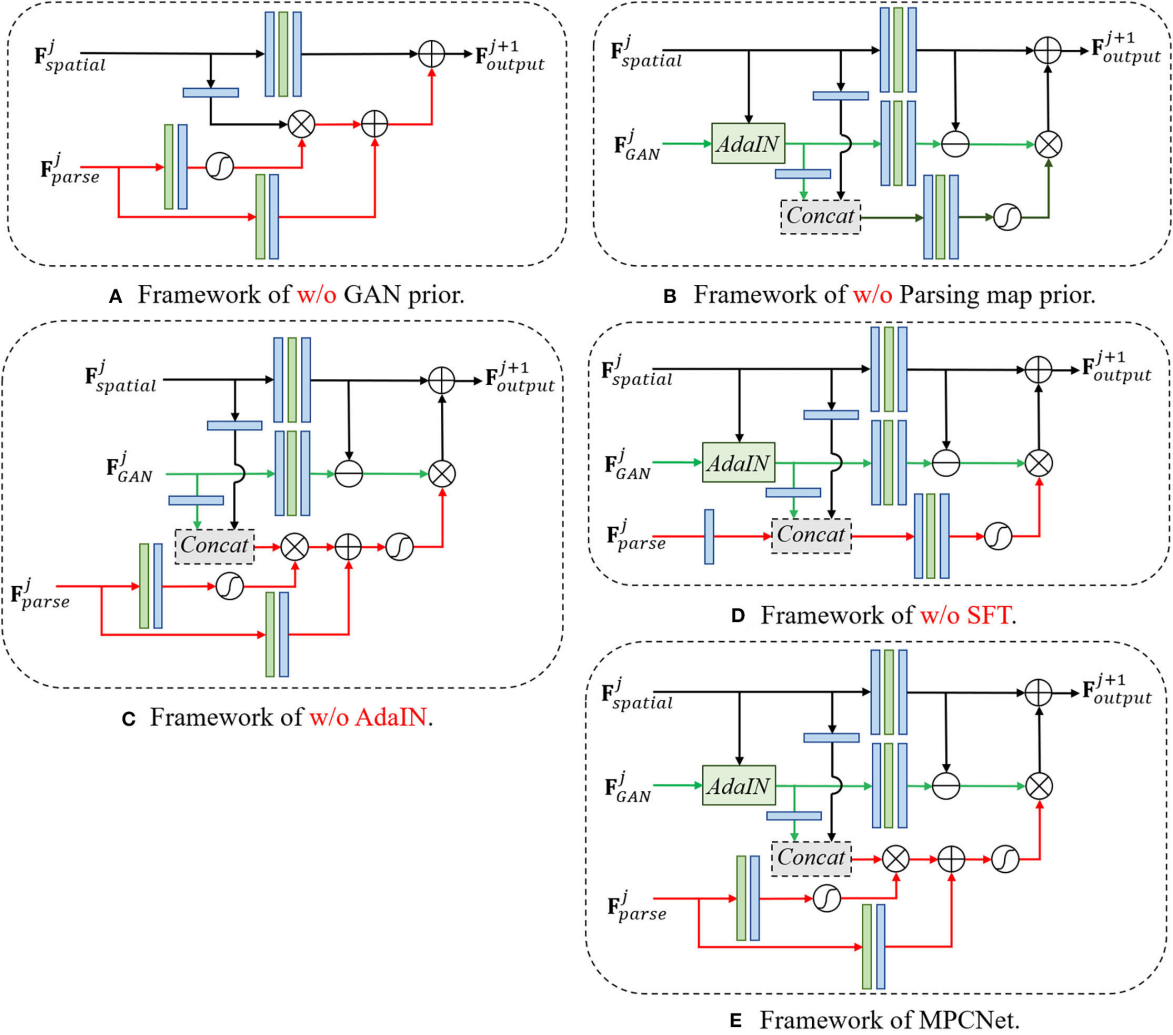
Structure diagrams of different network versions used in the ablation study. **(A)** Framework of w/o GAN prior. **(B)** Framework of w/o parsing map prior. **(C)** Framework of w/o AdaIN. **(D)** Framework of w/o SFT. **(E)** Framework of w/o MPCNet.

We further conduct an ablation study to verify the superiority of our multi-prior collaboration framework (see Figure 6). To demonstrate the superiority of our prior-integration method, we remove used modules separately and visualize some comparison results of different variants. The characteristics of different model variants used in the ablation study are summarized in Table 1.

**Table 1:** Summary of model characteristics presented in the ablation study.

**Configuration**	**GAN prior**	**AdaIN**	**Parsing map prior**	**SFT**
w/o GAN prior	×	×	√	√
w/o Parsing map prior	√	√	×	×
w/o AdaIN	√	×	√	√
w/o SFT	√	√	√	×
MPCNet (ours)	√	√	√	√

**Figure 7:**
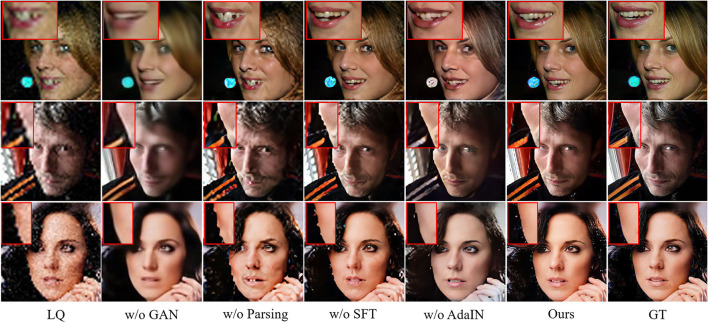
Qualitative comparison of the effect of using different components to form the Blind Face Restoration (BFR) networks. Viewed best when zoomed in.

**Pretrained GAN prior**: *w/o GAN prior* denotes the basic model that consists of the decoder part of U-shaped DNN which leverages the encoded intermediate spatial features and parsing map prior priors to restore the HQ face, during which the generative priors are abandoned. This model is in essence equivalent to a parsing map priors guided face restoration network and is included here to demonstrate the importance of generative priors. As the comparison between MPCNet and w/o GAN prior shown in Figure 7 and Table 2, it is evident that the GAN priors can provide diverse and rich facial details for our BFR task.

**Table 2:** The quantitative performance of different variants on CelebA-Test.

**Configuration**	**FID ↓**	**LPIPS ↓**	**PSNR ↑**	**SSIM ↑**
w/o GAN prior	120.14	0.5576	22.13	0.6165
w/o Parsing map prior	45.51	0.4133	21.54	0.6415
w/o AdaIN	29.49	0.3173	23.50	0.6629
w/o SFT	37.88	0.3838	22.64	0.6582
MPCNet (ours)	29.26	0.3097	23.82	0.6684

**Pretrained parsing map prior**: *w/o Parsing map prior* denotes the model that consists of the decoder part of U-shaped DNN which leverages the encoded intermediate spatial features and generative priors to restore the HQ face, during which the parsing map prior are abandoned. This model is in essence equivalent to a generative priors guided face restoration network and is included here to demonstrate the importance of parsing map priors. As the comparison between MPCNet and w/o Parsing map prior shown in Figure 7 and Table 2, it is evident that the Parsing map priors can provide the geometry and semantic information for covering the shortage of GAN priors and further improve the fidelity of restored face image.

**AdaIN**: *w/o AdaIN* denotes the model that consists of the decoder part of U-shaped DNN which leverages the encoded intermediate spatial features with types of facial priors to restore the HQ face, during which the AdaIN is abandoned. This model is included here to demonstrate the importance of AdaIN. As the comparison between MPCNet and w/o AdaIN shown in Figure 7 and Table 2, it is evident that the AdaIN module can translate the content features to the desired style with effect and, thus, makes the illumination condition of restored face consistent with the original input.

**Spatial feature transform**: *w/o SFT* denotes the model that consists of the decoder part of U-shaped DNN which leverages the encoded intermediate spatial features with types of facial priors to restore the HQ face, during which the SFT is abandoned. This model is included here to demonstrate the importance of SFT. As the comparison between MPCNet and w/o SFT shown in Figure 7 and Table 2, it is evident that the SFT module can make full use of the parsing map priors to guide the face restoration branch to pay more attention to the essential facial parts reconstruction.

### 4.4. Comparison With the State-Of-The-Art

#### 4.4.1. Comparison of Synthetic Dataset for BFR

To quantitatively compare MPCNet with other state-of-the-arts methods: WaveletSRNet (Huang et al., 2017), Super-FAN (Bulat and Tzimiropoulos, 2018), DFDNet (Li et al., 2020a), HiFaceGAN (Yang et al., 2020), PSFRGAN (Chen et al., 2021), and GPEN (Yang et al., 2021), we first perform experiments on synthetic images. Following the comparison experiments setting in Yang et al. (2021), we directly compared with these state-of-the-arts models trained by the original authors in the experiments. Except for Super-FAN, we adopt their official codes and finetune them on our face training set for fair comparisons. Table 3 lists the perceptual metrics (FID and LPIPS) and pixel-wise metrics (PSNR and SSIM) results on the CelebA-Test testset. It can be seen that our MPCNet achieves comparable PSNR and SSIM indices to other competing methods, but it achieves significant performance gains over all the competing methods on FID and LPIPS indices, which are better measures than PSNR for the face image perceptual quality.

**Table 3:** Quantitative comparison on CelebA-Test for blind face restoration (BFR).

**Methods**	**FID ↓**	**LPIPS ↓**	**PSNR ↑**	**SSIM ↑**
Input	158.72	0.6185	20.23	0.6823
WaveletSRNet	119.75	0.5351	22.87	0.6451
Super-FAN	100.23	0.5085	22.15	0.6377
DFDNet	43.74	0.3926	21.84	0.6439
HiFaceGAN	64.37	0.4795	21.05	0.5444
PSFRGAN	31.92	0.3226	23.17	0.6472
GPEN	31.41	0.3267	22.91	0.6428
MPCNet (ours)	29.26	0.3097	23.82	0.6684

**Figure 8:**
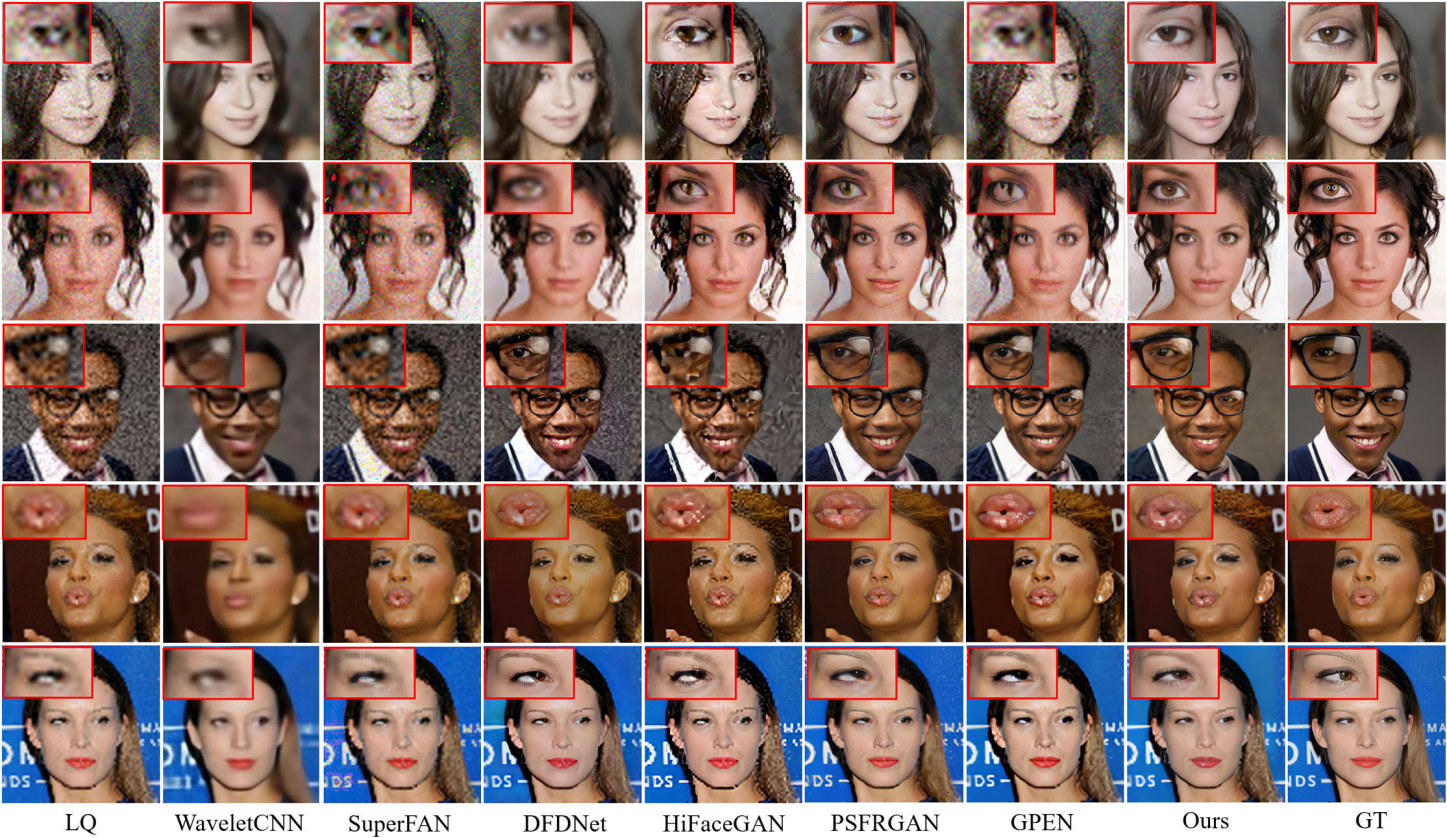
Comparison of qualitative performance with state-of-the-art BFR methods from the literature.

Figure 8 compares the BFR results on some degraded face images by the competing methods. One can see that the competing methods fail to produce reasonable face reconstructions. They tend to generate over-smoothed face images with distorted facial structures. Due to the powerful generative facial prior, it is obvious that our MPCNet is more effective in restoring fine details while suppressing visual artifacts. In comparison with the competing methods, the results by MPCNet are visually photo-realistic and can correctly recover finer and identity-aware details, especially in eyes, nose, and mouth regions.

#### 4.4.2. Experiments on Arbitrary Scales Face Super-Resolution (SR)

**Figure 9:**
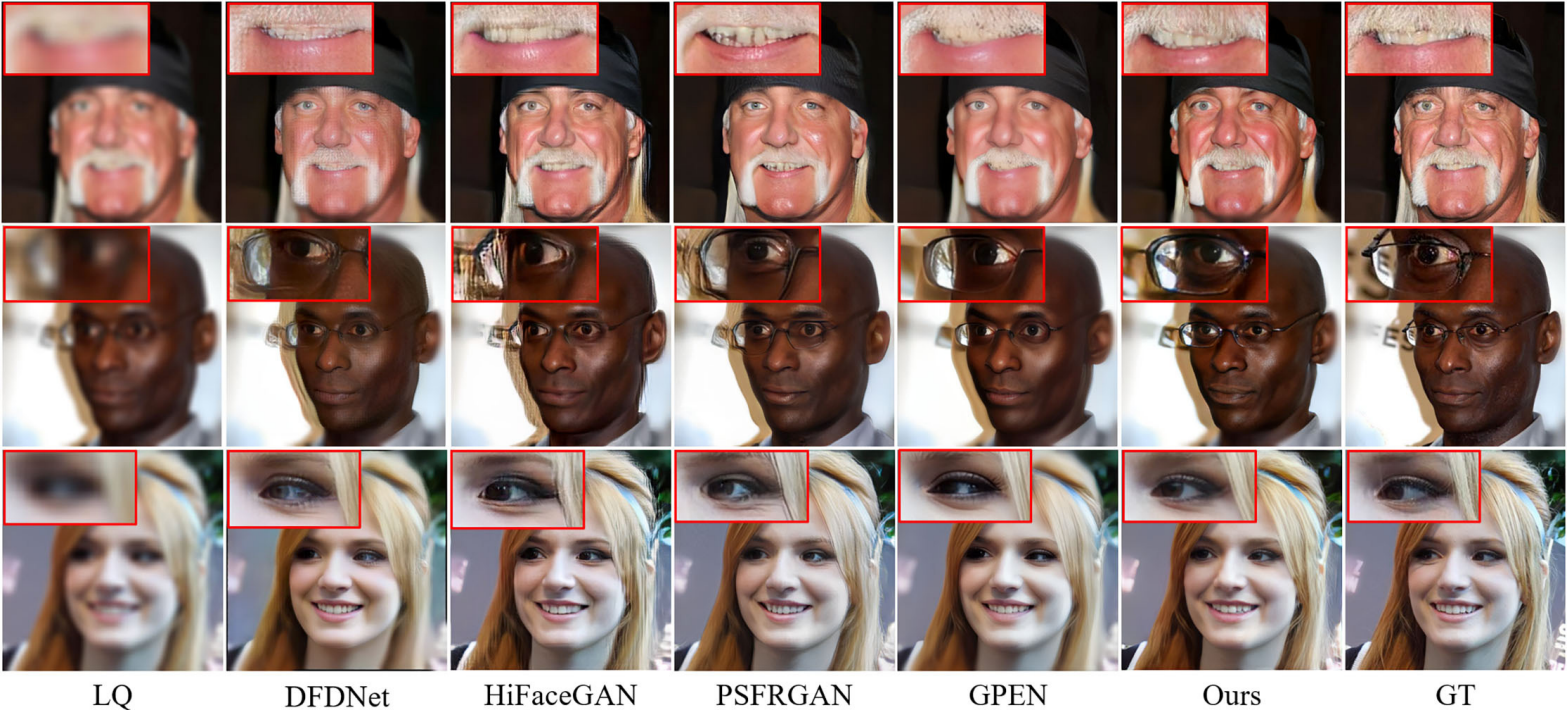
Visual comparison for non-integer face super-resolution (SR) (i.e., ×6.5 SR, kernel width = 7).

**Figure 10:**
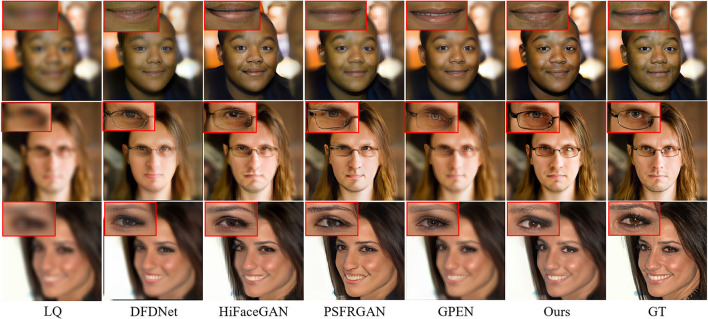
Visual comparison for non-integer face SR (i.e., ×7.15 SR, kernel width = 10).

We can see from Table 4 that our MPCNet achieves comparable performance to GPEN on all scale factors, with average PSNR improvements of 0.85. Compared to PSFRGAN, our MPCNet achieves notable performance improvements (23.98 vs. 23.24) for ×4 SR and (24.19 vs. 23.44) for ×7 SR. This clearly demonstrates that the proposed our MPCNet can enable scale-arbitrary SR without performance degradation on SR with fixed scale factors. Figures 9, 10 illustrate the qualitative SR results on two non-integral scale factors. As shown in these zoom-in regions, we can see that our MPCNet produces better visual results than other methods with fewer artifacts. For example, GPEN and PSFRGAN cannot recover the eyes and mouth regions reliably and suffer from obvious distorted artifacts. In contrast, our MPCNet produces finer details.

**Table 4:** Peak signal-to-noise ratio (PSNR) results achieved for ×4/ × 5/ × 6/ × 7/ × 8 face super-resolution (SR).

**Methods**	**Scale**				
	**×4**	**×5**	**×6**	**×7**	**×8**
Input	22.97	22.63	21.33	20.64	20.34
DFDNet	23.41	23.15	22.87	22.28	21.95
HiFaceGAN	23.12	22.85	22.68	22.19	21.17
PSFRGAN	24.52	24.14	23.92	23.44	23.24
GPEN	24.39	24.00	23.81	23.35	23.12
MPCNet (ours)	25.26	24.84	24.65	24.19	23.98

#### 4.4.3. Experiments on Different Types Blur Kernels Degradations

**Figure 11:**
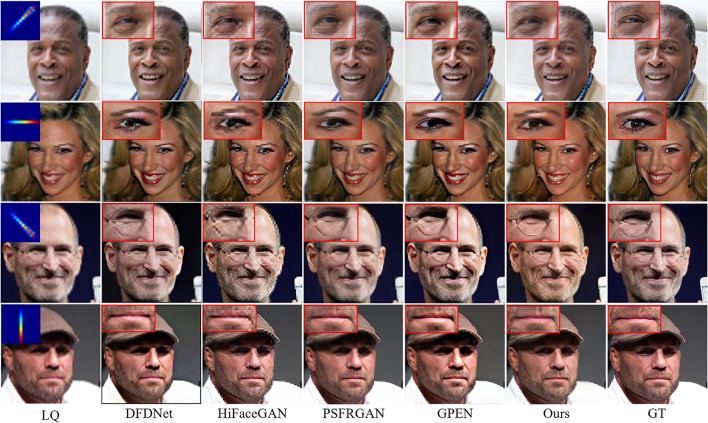
Visual comparison achieved on noise-free degradations with different blur kernels. The blur kernels are illustrated with green boxes.

We adopt 4 Gaussian blur kernels with different sizes and 4 motion blur kernels in four different directions to test the BFR performance of the competing methods. It can be observed from Table 5 that HiFaceGAN produces relatively low performance on complex degradations. Since HiFaceGAN is sensitive to degradation estimation errors, its performance for complex degradations is limited. By incorporating the prior features from pretrained face synthesis GAN and face parsing network in an adaptive and progressive manner, our MPCNet exhibits good generalization on complex degradations. Figure 11 further illustrates the visualization results produced by different methods. Our MPCNet achieves much better visual quality while other methods suffer obvious blurring artifacts.

**Table 5:** Peak signal-to-noise ratio results achieved on noise-free degradations with different blur kernels.

**Method**	**Blur kernel**							
	** 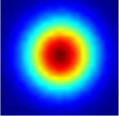 **	** 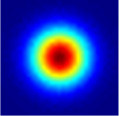 **	** 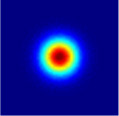 **	** 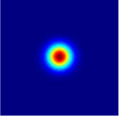 **	** 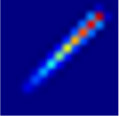 **	** 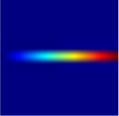 **	** 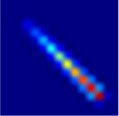 **	** 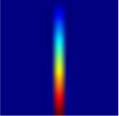 **
Input	20.34	20.81	21.02	21.49	21.17	21.45	21.23	21.16
DFDNet	20.83	21.54	21.79	22.18	21.98	22.64	22.47	22.74
HiFaceGAN	20.41	21.19	21.65	21.89	21.64	22.41	22.21	22.49
PSFRGAN	22.56	22.92	23.14	23.58	23.12	23.09	22.76	23.27
GPEN	22.15	22.44	22.85	23.29	23.10	23.02	22.88	23.15
MPCNet (ours)	23.04	23.36	23.59	23.97	23.57	23.43	23.25	23.48

*The kernel widths are set to 10*.

#### 4.4.4. Experiments on Different Levels Noises Degradations

We set 6 noise levels to evaluate the restoration performance of the competing methods. In Table 6, we present the PSNR numbers for all noise levels. Since each APFF block can integrate generative priors and parsing maps priors to generate the fusion feature maps for guiding face restoration, when applying to complicated degradation scenarios, the fusion feature maps can correctly find where to incorporate guidance prior features in an adaptive manner, making our MPCNet outperform all the competitive algorithms for all noise levels.

**Table 6:** Peak signal-to-noise ratio results achieved on CelebA-Test degraded by different level noises.

**Methods**	**Noise**					
	**0**	**5**	**10**	**15**	**20**	**25**
Input	21.54	21.25	20.91	20.67	20.45	20.21
DFDNet	22.26	21.94	21.77	21.39	21.08	20.70
HiFaceGAN	21.93	21.59	21.25	20.98	20.74	20.33
PSFRGAN	23.66	23.50	23.38	23.06	22.79	22.42
GPEN	23.31	23.17	22.94	22.78	22.31	22.09
MPCNet (ours)	24.03	23.81	23.65	23.43	23.24	22.95

**Figure 12:**
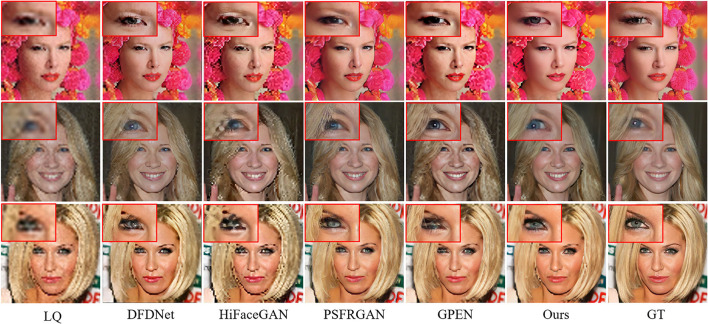
Visual comparison achieved on CelebA-Test with the noise level set at 5.

**Figure 13:**
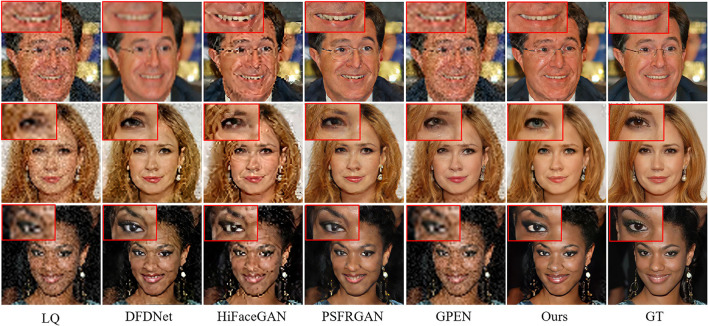
Visual comparison achieved on CelebA-Test with the noise level set at 15.

Figures 12, 13 present the visual comparison outperforms all the other techniques published in Table 6 and produces the best perceptual quality images. The closer inspections on the eyes, nose, and mouth regions reveal that our network generates textures closest to the ground-truth with fewer artifacts and more details for all noise levels.

#### 4.4.5. Comparison of Real World LQ Images

**Figure 14:**
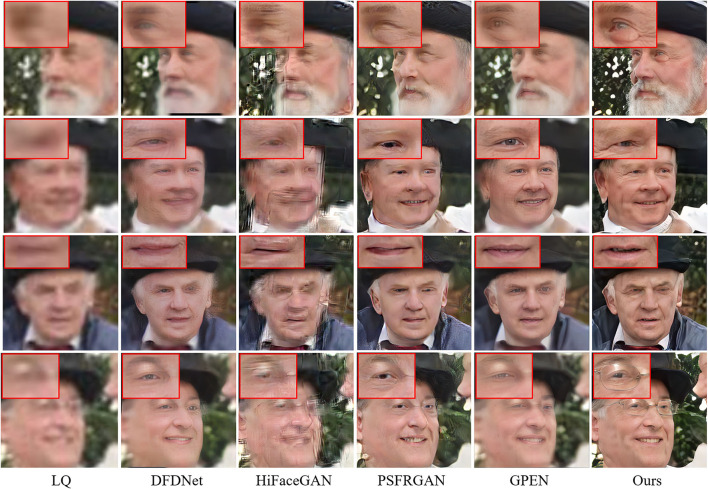
Visual comparisons of competing methods with top performance on real-world low-quality (LQ) images (×21.4 SR).

**Figure 15:**
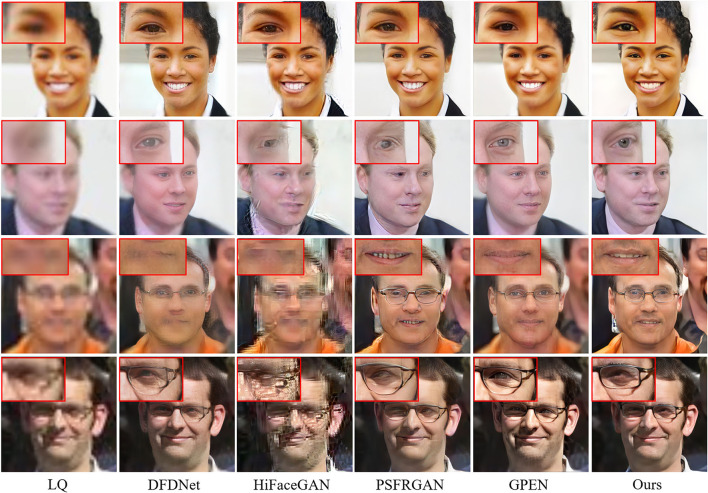
Visual comparisons of competing methods with top performance on real-world LQ images (×14.2 SR).

To test the generalization ability, we evaluate our model on the real-world dataset. The quantitative results are shown in Table 7. Our MPCNet achieves superior performance and shows its remarkable generalization capability. Although GPEN also obtains comparable perceptual quality, it still fails in recovering the faithful face details as shown in Figures 14, 15.

**Table 7:** Quantitative comparison on the real-test.

**Methods**	**NIQE ↓**	**FID ↓**
Input	14.623	183.73
DFDNet	5.824	107.36
HiFaceGAN	6.141	124.12
PSFRGAN	5.783	94.51
GPEN	5.597	91.63
MPCNet (ours)	4.849	89.18

The qualitative comparisons are shown in Figures 14, 15. The cropped LR face images from real-world images in Figures 14, 15 are 24 × 24 pixels and 36 × 36 pixels, and then we rescale the LR images to a fixed input size for MPCNet of 512 × 512 pixels. Thus, the scale factors of the visual comparisons are 21.4× and 14.2×, respectively. MPCNet seamlessly integrates the advantages of generative priors and face-specific geometry priors for restoring real-life photos with faithful facial details. Since the generative priors can provide adequate details and the parsing map priors provide geometry and semantic information, our method could produce plausible and realistic faces on complicated real-world degradation while other methods fail to recover faithful facial details or produce artifacts. Not only can our method perform well in common facial components like mouth and nose, but it can also perform better in hair and ears, as the parsing map priors can take the whole face into consideration rather than separate parts.

## 5. Conclusion

We have proposed a MPCNet to seamlessly integrate the advantages of generative priors and face-specific geometry priors. Specifically, we pretrained an HQ face synthesis GAN and a parsing mask prediction network and then embedded them into a U-shaped DNN as decoder priors to guide face restoration, during which the generative priors can provide adequate details and the parsing map priors provide geometry and semantic information. By designing an adaptive priors feature fusion (APFF) block to incorporate the prior features from pretrained face synthesis GAN and face parsing network in an adaptive and progressive manner, our MPCNet exhibited good generalization in a real-world application. Experiments demonstrated the superiority of our MPCNet in comparison to state-of-the-arts and also showed its potential in handling real-world LQ images from several practical applications.

## Data Availability Statement

The original contributions presented in the study are included in the article/supplementary material, further inquiries can be directed to the corresponding author.

## Author Contributions

ZT: conceptualization, methodology, software, writing–original draft preparation, and data curation. XY: software, validation, visualization, and supervision. CW: investigation, writing–reviewing, and editing. All authors contributed to the article and approved the submitted version.

## Funding

This work was supported in part by the National Natural Science Foundation of China (under Grant Nos. U20A20197 and 61973063), Liaoning Key Research and Development Project (2020JH2/10100040), Natural Science Foundation of Liaoning Province (2021-KF-12-01), and the Foundation of National Key Laboratory (OEIP-O-202005).

## Conflict of Interest

The authors declare that the research was conducted in the absence of any commercial or financial relationships that could be construed as a potentialconflict of interest.

## Publisher's Note

All claims expressed in this article are solely those of the authors and do not necessarily represent those of their affiliated organizations, or those of the publisher, the editors and the reviewers. Any product that may be evaluated in this article, or claim that may be made by its manufacturer, is not guaranteed or endorsed by the publisher.
